# Capability of Neutrophils to Form NETs Is Not Directly Influenced by a CMA-Targeting Peptide

**DOI:** 10.3389/fimmu.2017.00016

**Published:** 2017-01-27

**Authors:** Christian Maueröder, Nicolas Schall, Frédéric Meyer, Aparna Mahajan, Benjamin Garnier, Jonas Hahn, Deborah Kienhöfer, Markus H. Hoffmann, Sylviane Muller

**Affiliations:** ^1^Department of Internal Medicine 3 – Rheumatology and Immunology, Friedrich-Alexander-University Erlangen-Nürnberg (FAU), Universitätsklinikum Erlangen, Erlangen, Germany; ^2^CNRS, Immunopathology and Therapeutic Chemistry/Laboratory of Excellence MEDALIS, Institut de Biologie Moléculaire et Cellulaire, Strasbourg, France; ^3^University of Strasbourg Institute for Advanced Study (USIAS), Strasbourg, France

**Keywords:** NET formation, neutrophils, autophagy, murine models of lupus, systemic lupus erythematosus, P140/Lupuzor

## Abstract

During inflammatory reaction, neutrophils exhibit numerous cellular and immunological functions, notably the formation of neutrophil extracellular traps (NETs) and autophagy. NETs are composed of decondensed chromatin fibers coated with various antimicrobial molecules derived from neutrophil granules. NETs participate in antimicrobial defense and can also display detrimental roles and notably trigger some of the immune features of systemic lupus erythematosus (SLE) and other autoimmune diseases. Autophagy is a complex and finely regulated mechanism involved in the cell survival/death balance that may be connected to NET formation. To shed some light on the connection between autophagy and NET formation, we designed a number of experiments in human neutrophils and both in normal and lupus-prone MRL/lpr mice to determine whether the synthetic peptide P140, which is capable of selectively modulating chaperone-mediated autophagy (CMA) in lymphocytes, could alter NET formation. P140/Lupuzor™ is currently being evaluated in phase III clinical trials involving SLE patients. Overall our *in vitro* and *in vivo* studies established that P140 does not influence NET formation, cytokine/chemokine production, or CMA in neutrophils. Thus, the beneficial effect of P140/Lupuzor™ in SLE is apparently not directly related to modulation of neutrophil function.

## Introduction

Systemic lupus erythematosus (SLE) is an autoimmune disorder characterized by chronic reactivity against components of the cell nucleus that results in the formation of antinuclear autoantibodies and multiorgan involvement. It is thought that the immune system in lupus is sensitized to intracellular antigens following extended exposure to chromatin under inflammatory conditions. This can be caused by insufficient removal of cells having undergone apoptosis or the formation of neutrophil extracellular traps (NETs) (1–3). NETs contain a complex network made of processed chromatin bound to granular and selected cytoplasmic proteins. Due to the large amounts of nuclear antigens freely accessible in NETs, they are prime candidates for the initiation or enhancement of autoimmunity and organ damage in lupus (4). Blockade of NET formation by pharmacological inhibition of peptidylarginine deiminases, which are essential for certain forms of NET formation (5), is therefore considered a promising strategy to ameliorate the clinical course of SLE (6, 7).

There is currently no cure for lupus but in the list of molecules that show clear benefits in patients with SLE are antimalarials, such as hydroxychloroquine/Plaquenil™, a therapeutic that is generally prescribed in combination with steroids or other compounds. This molecule, which unfortunately induces undesirable side effects that can be dramatic (retinopathy), directly influences lysosomal pathways. Other drugs acting at the level of the lysosome through known or assumed modulation of autophagic pathways include rapamycin, bortezomib/Velcade™ (8), 15-deoxyspergualin/Gusperimus™ that however displays serious adverse effects (most notably leukopenia), and P140/Lupuzor™ (9–11). The P140 peptide was shown to ameliorate lupus in the MRL/lpr murine model of the disease and to significantly delay mortality (12, 13). Moreover, treatment of patients with SLE with P140/Lupuzor™ convincingly improved the biological and clinical status of patients in a multicenter, randomized, placebo-controlled phase IIb trial and was considered efficacious and safe for the treatment of SLE (14). P140/Lupuzor™ recently entered into multicenter, double-blind, placebo-controlled phase III clinical trials in the US, Europe, and countries of the West Indian Ocean. The 21-mer peptide P140 encompasses residues 131–151 of the spliceosomal U1-70K protein, containing a phosphoserine at position 140 (hence its name). In MRL/lpr mice, P140 was shown to work via inhibition of autophagy, particularly chaperone-mediated autophagy (CMA), which we discovered to be hyperactivated in lymphocytes in this mouse model (10, 11, 15). In the lysosomes of MRL/lpr B cells, the phosphorylated peptide P140, but interestingly not the non-protective unphosphorylated peptide (termed 131-51), is supposed to compromise CMA by disruption of the lumenal HSPA8 heterocomplexes containing HSP90 as it does in vitro (11). This inhibitory effect on CMA modulates autoantigen loading to MHCII molecules and therefore results in a diminished priming of autoreactive T cells. Consequentially, the proliferation of autoreactive B cells and their differentiation into deleterious autoantibody-secreting plasma cells is reduced.

Previous studies have stated that phorbol myristate acetate (PMA)-induced formation of NETs requires autophagy (16). Autophagy was also claimed to be involved in various neutrophil functions and in neutrophil-mediated inflammation (17, 18). We therefore wondered whether the effect of P140/Lupuzor™ on lupus features could be related to an influence on neutrophil function.

## Materials and Methods

### Peptides

P140, scrambled P140 (ScP140), and the non-phosphorylated peptide 131-151 were synthesized and purified as described previously (19). Homogeneity of peptides was checked by analytical high-performance liquid chromatography, and their identity was assessed by MALDI-TOF mass spectrometry.

### Mice

C3H/HeOuJ (thereafter named C3H) and MRL/MpJ-*Fas^lpr^*/2J (thereafter named MRL/lpr) mice were bought from Charles River. MRL/lpr mice were also kindly given by Prof. Lars Nitschke, Division of Genetics, Department of Biology, Erlangen-Nuremberg University. Mice were kept at 12 h light/dark cycles in polystyrene cages in the animal facilities of the University of Erlangen-Nuremberg and CNRS in Strasbourg and were fed with standard rodent chow and water *ad libitum*. Experiments, which were performed with the investigators blinded to group allocation, were approved by the local ethical committees (Regierung von Unterfranken, Germany, and Comité Régional d’Ethique en Matière d’Expérimentation Animale de Strasbourg, respectively).

### *In Vivo* Treatment with Peptides

P140, ScP140, and 131-51 peptides were resuspended in distilled water to a concentration of 10 mg/mL and further diluted in 0.9% (w/v) NaCl to a concentration of 100 µg/100 µl. Each mouse received two intravenous injections of 100 µg P140 or control peptides at days 1 and 4. Twenty-four hours after the last injection, blood was collected in heparinized collection tubes (Sarstedt) and processed and described as below.

### Isolation of Human and Mouse Neutrophils

A total of 20 mL heparinized blood were taken from each human normal healthy donor. A total of 15 mL of phosphate-buffered saline (PBS) without calcium and magnesium (Thermo Fisher Scientific) were added and polymorphonuclear leukocytes (PMNs) were isolated by standard density gradient centrifugation using Ficoll (Bio-Rad). To remove contaminating erythrocytes, PMNs were subjected to short cycles of hypotonic lysis with deionized water. Finally, PMNs were adjusted to a concentration of 2 × 10^6^ cells/mL in PBS without calcium and magnesium (Thermo Fisher Scientific).

For isolation of mouse neutrophils, single-cell suspensions were prepared from spleens of mice by squeezing through a 70 µm cell strainer. After hypotonic lysis of erythrocytes, neutrophils were isolated by negative selection using the EasySep™ mouse neutrophil enrichment kit (Stemcell Technologies) according to the manufacturer’s instruction.

Purity of isolated neutrophils was checked by flow cytometry and was above 95% (human neutrophils) or 85% (mouse neutrophils), respectively. All experiments were approved by the ethical committee of the University of Erlangen-Nuremberg.

### Plate Reader-Based Quantification of NET Formation

Isolated neutrophils were adjusted to a concentration of 2 × 10^6^ cells/mL in Hanks’ balanced salt solution (HBSS; Thermo Fisher Scientific). Cell suspension (100 µL) was pipetted into each well of a 96-well cell plate. A total of 100 μL of HBSS and 5 µM Sytox Green (Thermo Fisher Scientific) containing either PMA (200 ng/mL; Sigma), ionomycin (2 µg/mL; Sigma), or vehicle control were added to the cells. The plate was tightly sealed and analyzed in an infinite^®^ 200 pro plate reader (TECAN). Relative fluorescence units were normalized to the starting values and the respective vehicle control.

### Immunohistochemical Analysis of NET Formation

Isolated neutrophils were adjusted to a concentration of 2 × 10^6^ cells/mL in HBSS containing calcium and magnesium. A total of 100 μL of cell suspension was added to each well of an 8-well cell chamber slide (Thermo Fisher Scientific). A total of 100 μL of HBSS containing either 200 ng/mL PMA, 2 µg/mL ionomycin, or vehicle control were added to the cells. The chamber slide was incubated at 37°C and 5% CO_2_ for 2 h. Subsequently, 1% (v/v) paraformaldehyde (Merck) was added to each well and the preparations incubated for 18 h at 4°C. Samples were blocked with 10% (v/v) fetal calf serum (FCS; Biochrome)/2% (w/v) bovine serum albumin (BSA) in PBS for 1 h at room temperature. Primary antibody for neutrophil elastase (NE) (Abcam ref. ab21595; 1:200) or citrullinated histone H3 (Abcam ref. ab1503; 1:100) were added in 10% FCS/2% BSA in PBS for 18 h at 4°C. Slides were washed three times with PBS and secondary Cy5-conjugated anti-rabbit IgG antibody (Jackson ImmunoResearch) was added for 1.5 h at room temperature in the dark. Slides were washed with PBS. Staining solution containing 2.5 µM SYTOX Green in PBS was added for 15 min at room temperature. Slides were washed with H_2_O and samples were embedded in mounting medium (BIOZOL). Slides were analyzed the same day on a BZ-X710 microscope (Keyence). Events positive for SYTOX Green were analyzed with regard to area and mean intensity by Photoshop CS5 software. Percentage of NETs was defined as PI/NE double-positive events with >3-fold mean nuclear size on three random slide sections.

### Analysis of Autophagy

Spleens were homogenized through a 70 µM strainer, washed, and seeded at a concentration of 4 × 10^6^ cells/well in a 48-well plate with 1 mL of complete culture media in the presence or absence of lysosomal protein inhibitors pepstatin A (5 µg/mL; ref. P5318; Sigma) and E64d (5 µg/mL; ref. E8640; Sigma). After 4 h at 37°C under 5% CO_2_, cells were washed, lysed by adding 160 µL/sample of Laemmli buffer (ref. 161-0737; Bio-Rad) containing 5% (v/v) β-mercaptoethanol, and finally boiled at 95°C (5 min) before loading (20 µL samples, equivalent to 0.5 × 10^6^ cells) on 4–20% SDS-PAGE gradient gels for analysis. For western immunoblotting, the following antibodies were used: rabbit SQSTM1/p62 (0.5 µg/mL; ref. ab109012), rabbit HSPA8 (0.5 µg/mL; ref. ab51052), and rabbit ATG12/5 (ref. ab155589), all from Abcam, and mouse microtubule-associated-protein light chain 3b (MAP1LC3B) (0.5 µg/mL; ref. M186-3) from MBL. Secondary antibodies were peroxidase-conjugated goat anti-mouse IgG Fc (50 ng/mL; ref. 115-035-008) and goat anti-rabbit IgG Fc (25 ng/mL; ref. 111-035-008) from Jackson ImmunoResearch. ACTB-reacting antibodies were from Santa Cruz (10 ng/mL; ref. sc47778).

### Measurement of Reactive Oxygen Species (ROS) and Inflammatory Mediators

For measurement of ROS in human PMNs, lithium-heparinized blood was incubated with dihydrorhodamine 123 (3 µg/mL, Molecular Probes) for 15 min at 37°C. Cells were then stained with anti-human CD14-eFluor450 and CD16-APC/Cy7, and ROS production was measured after incubation with peptides (10, 20, or 30 µM) or PMA (100 ng/mL) for 15 min at 37°C. Before analysis on a Beckman Coulter Gallios™, FACS samples were subjected to hypotonic water lysis.

Cytokines/chemokines in supernatants of human PMNs after 18 h incubation with varying concentrations of peptides, 2.5 µg/mL LPS, or 1 µM ionomycin in RPMI medium including 1% autologous serum were analyzed by Legendplex bead technology (BioLegend) and quantified on a Gallios™ cytofluorometer (Beckman Coulter).

### Statistical Analysis

For calculation of statistical differences, we used Mann–Whitney *U* test or unpaired Student’s *t*-test with Welch’s correction, Dunnett’s, or Bonferroni’s *post hoc* test, where applicable. Adjusted *p* < 0.05 was considered statistically significant. Computations and charts were performed using the GraphPad Prism 6 software.

## Results

**Figure 1:**
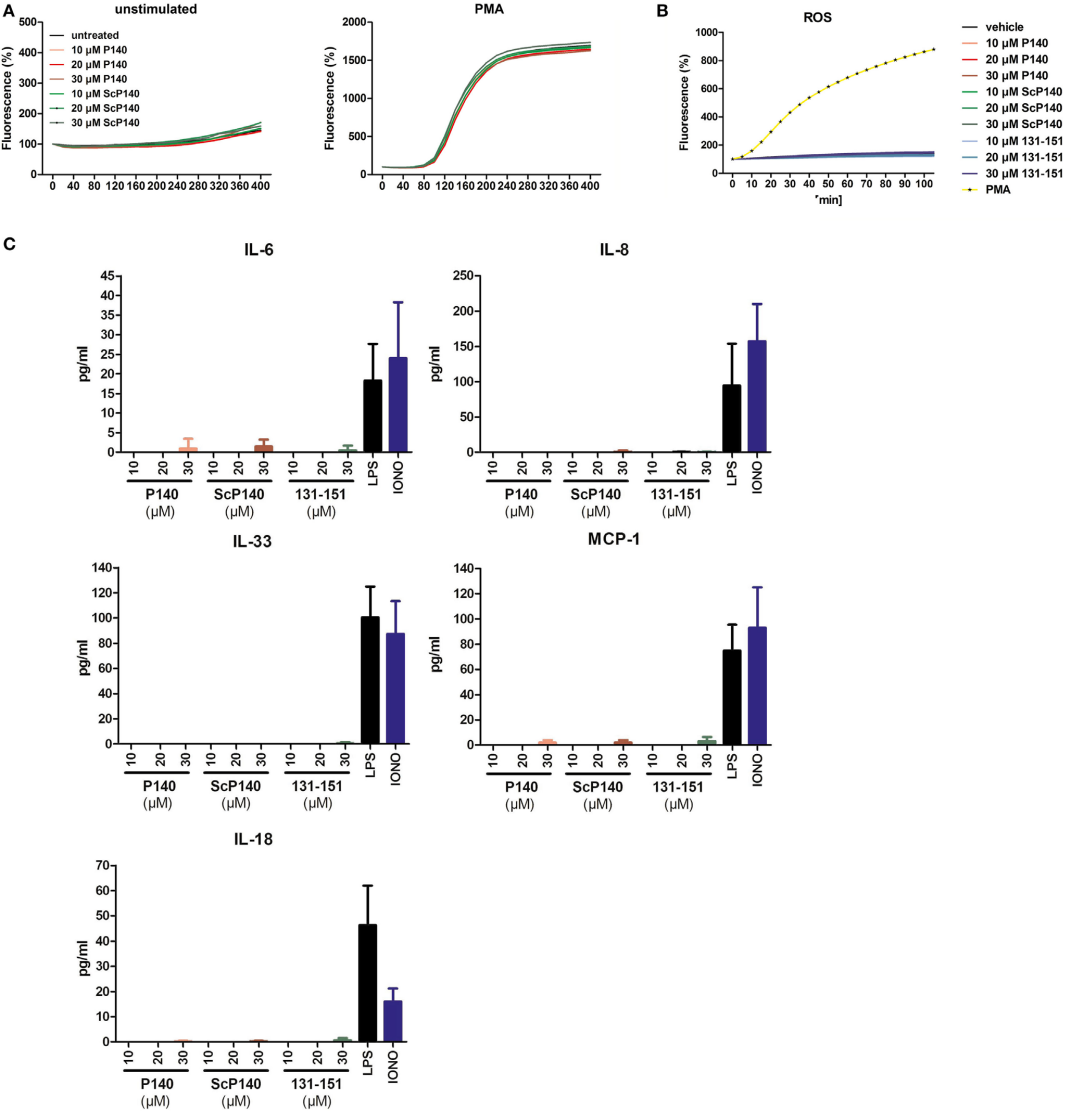
**No influence of P140 or the control peptides scrambled P140 (ScP140) and 131-51 on neutrophil extracellular trap (NET) formation or production of reactive oxygen species (ROS) or cytokines/chemokines in human PMN**. **(A)** Kinetics of relative SYTOX Green fluorescence in isolated human PMN after incubation with or without 100 ng/mL PMA. Graphs show data from one representative out of three normal healthy blood donors. **(B)** Intracellular ROS production or **(C)** cytokine/chemokine release into the supernatants upon incubation of isolated human PMN with varying concentrations of P140 or the control peptides ScP140 or 131-151. Baseline values of vehicle-treated neutrophils are subtracted. Bars in **(C)** show the means and SD of one representative out of two experiments.

To investigate a potential direct effect of P140 on NET formation, we pre-incubated isolated human PMNs with increasing concentrations of P140 or control ScP140 peptide (10–30 µM) for 1 h and measured SYTOX Green fluorescence after treatment with the NET-inducing agent PMA. The concentrations of peptides used for incubation have been previously shown not to be toxic to cells, except that prolonged incubation with concentrations over 20 µM induces granzyme B-dependent apoptosis in specific T cell subsets (13). Quantitative analysis of NET formation was performed in an established plate reader-based assay. Treatment with P140 or ScP140 did not reveal any effect on the capacity of PMNs to undergo NETosis in response to PMA (Figure 1A). Also neither P140 nor ScP140 directly induced NET formation, production of ROS, or cytokine/chemokine production in these cells (Figures 1B,C).

**Figure 2:**
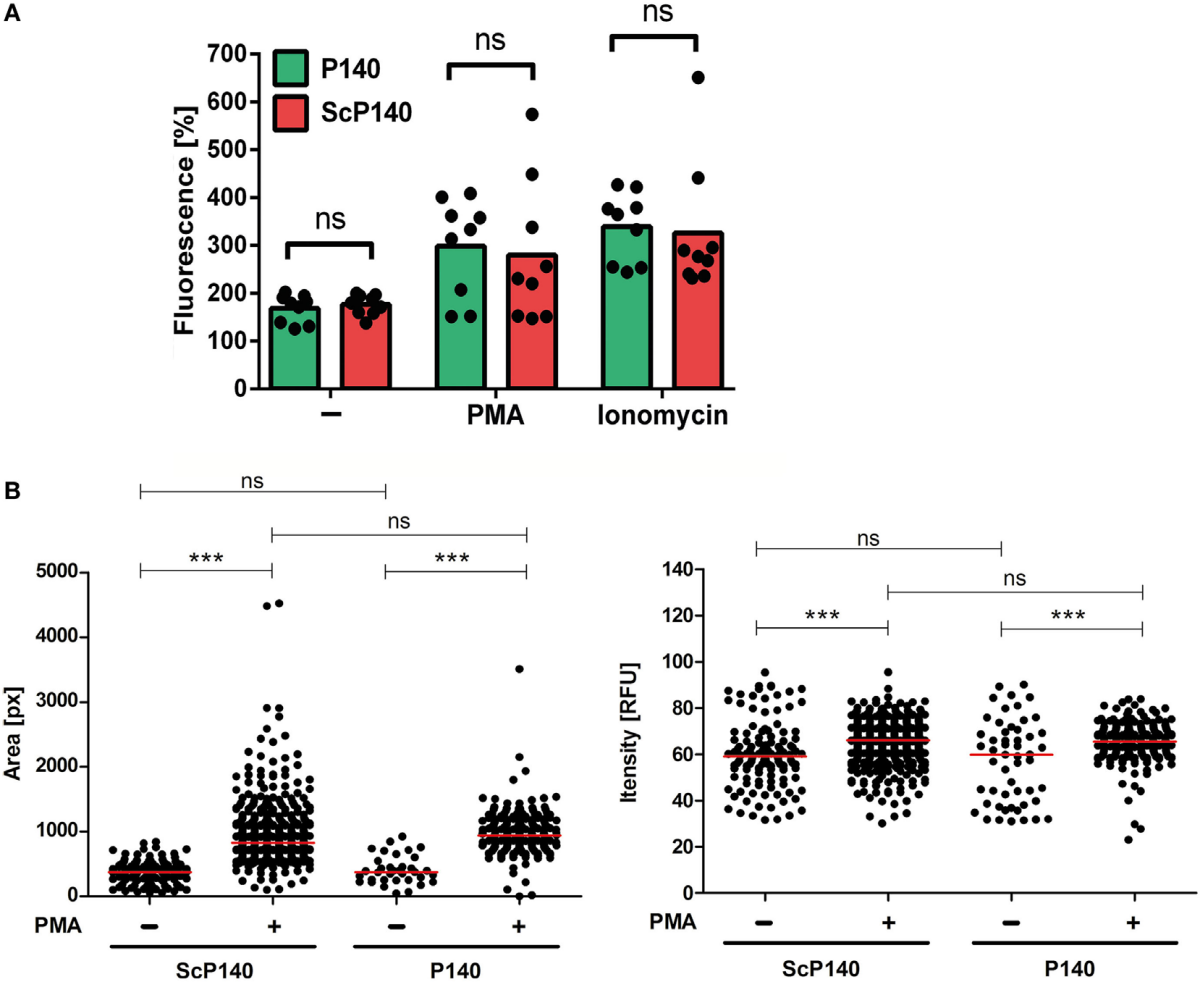
**Plate reader-based quantification of neutrophil extracellular trap formation in mice treated with P140 and scrambled P140 (ScP140)**. **(A)** Relative SYTOX Green fluorescence in neutrophils isolated from the blood of P140- or ScP140-pretreated mice incubated for 2 h with or without 100 ng/mL PMA or 2 µg/mL ionomycin. Graphs show individual values and means, one dot represents one mouse. ns, not significant. **(B)** Area and fluorescence intensity of SYTOX Green^+^ events in neutrophils isolated from the blood of P140- or ScP140-pretreated mice incubated for 2 h with or without 100 ng/mL PMA or 2 µg/mL ionomycin. Plots show medians and individual values from one representative out of 6–8 mice. ***p < 0.001, as determined by Student’s *t*-test with Bonferroni *post hoc* test. ns, not significant.

Isolated PMNs have a very limited lifespan, which precludes extended *in vitro* treatment with P140. To overcome this limitation, we administered two intravenous doses of 100 µg P140 or ScP140, respectively, into mice and sacrificed the mice 1 day later. Injections were performed in C3H mice because this strain harbors elevated number of neutrophils in the circulation (20), which resembles the human situation more closely than other mouse strains. Neutrophils from the blood were then purified from P140/ScP140-treated mice and NET formation was induced with PMA and ionomycin, respectively. Injection of P140 did not significantly influence the amount of total SYTOX fluorescence (Figure 2A). Also the mean area or fluorescence intensity of SYTOX Green^+^ events was not significantly different between cells isolated from P140 and ScP140-treated mice (Figure 2B).

**Figure 3:**
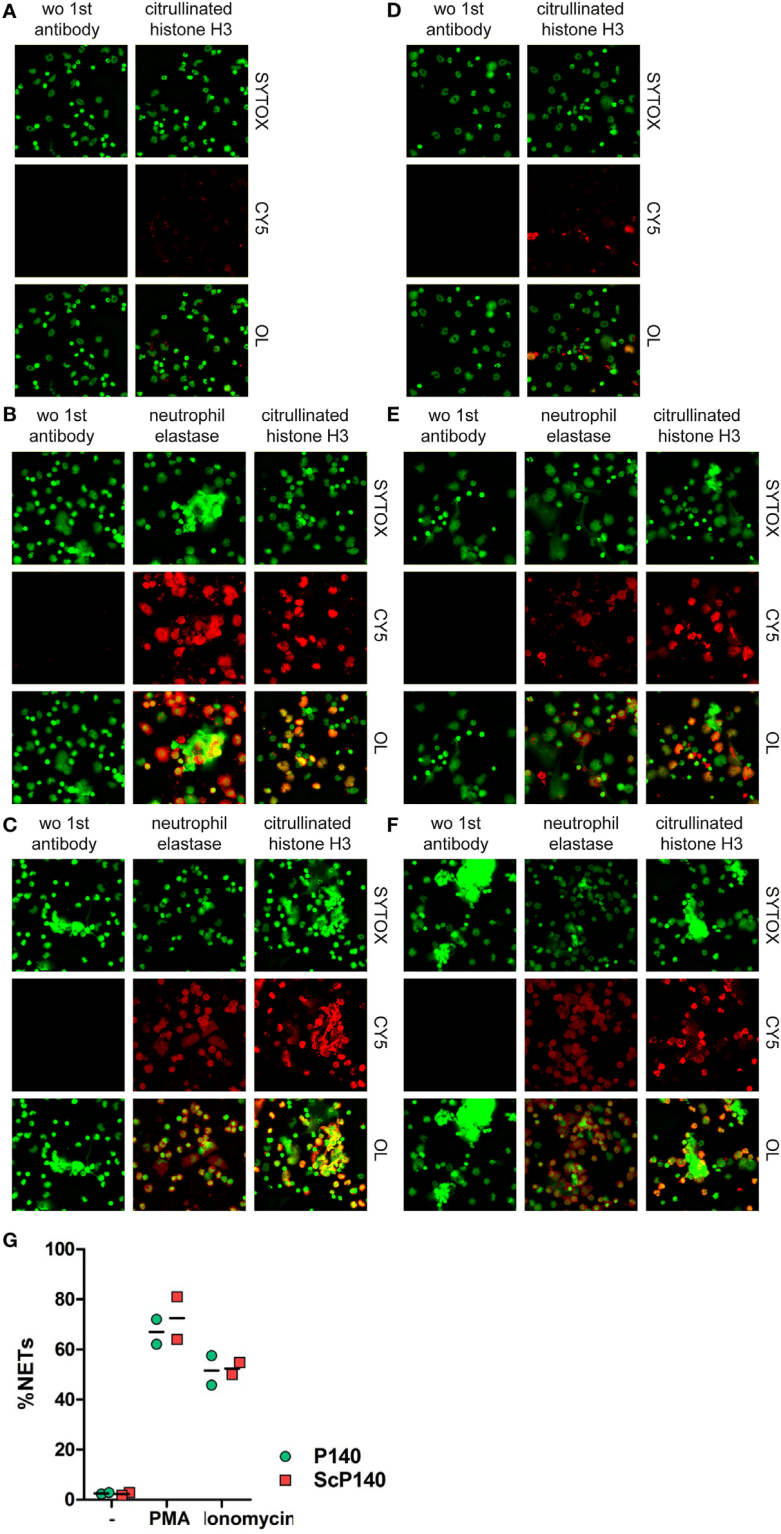
**Morphological analysis and quantification of neutrophil extracellular traps (NETs) in P140 and scrambled P140 (ScP140)-treated mice by fluorescence microscopy**. Representative immunofluorescence images of SYTOX Green-, neutrophil elastase (NE)-, and citrullinated histone H3 (citH3)-stained neutrophils isolated from ScP140- **(A–C)** and P140- **(D–F)** treated mice and incubated without external stimulus **(A,D)**, with PMA **(B,E)** or with ionomycin **(C,F)**. Left panels show staining controls incubated without (wo) primary antibody to NE or citH3. CY5 stands for control Cyanin 5 and OL stands for overlay. **(G)** Quantitative analysis of NETs. Scatter plots show individual values and mean of %NETs (defined as % PI^+^/NE^+^ cells with >3-fold mean nuclear size) from two mice.

SYTOX Green detects extracellular DNA, which can occur in NETs and other forms of cell death (21). However, DNA released during necrosis and other forms of cell death is not normally associated with material from neutrophil granules. NETs are defined by co-localization of nuclear content with granule proteins, such as myeloperoxidase, NE, or antimicrobial molecules (22). Furthermore, the citrullination of histones is typical for NET formation in response to some but not all triggers (5, 23, 24). We therefore performed a more thorough investigation of the morphological changes in PMA- and ionomycin-stimulated and unstimulated cells in neutrophils from mice pretreated with P140 or ScP140. This study was conducted by immunofluorescence microscopy for SYTOX Green, NE, and citrullinated histone H3. Irrespective of the marker that was followed, no significant differences were observed between the groups of C3H mice that had received the P140 and ScP140 peptides (Figure 3). Taken together, these results show that treatment with P140 does not affect NET formation in normal mice.

**Figure 4:**
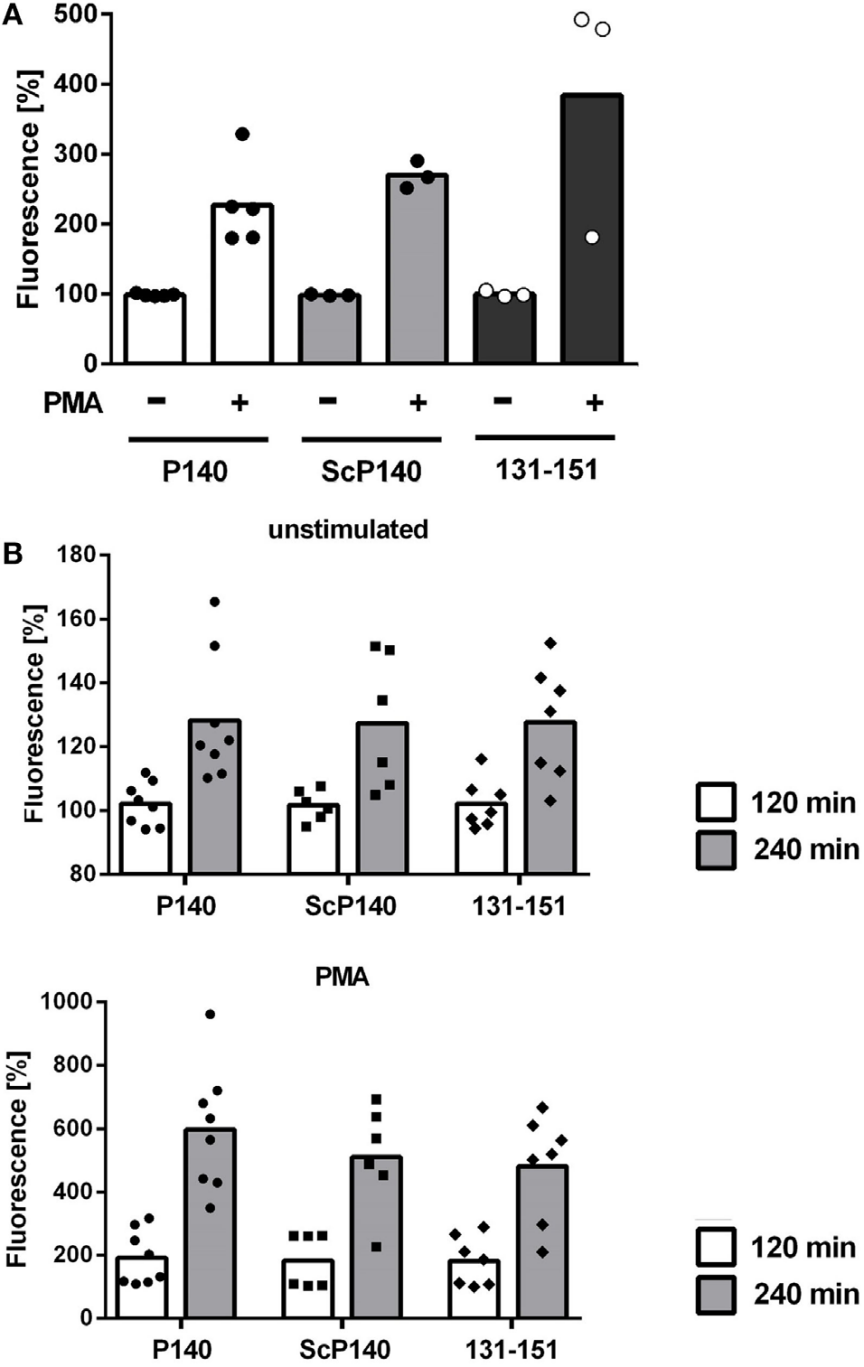
**Analysis of neutrophil extracellular trap formation in starved C3H and in MRL/lpr mice treated with P140, scrambled P140 (ScP140), or 131-151 peptides**. Neutrophils were isolated from the blood of C3H mice starved for 36 h **(A)** or MRL/lpr mice **(B)** pretreated with with injections of P140, ScP140, or the non-phosphorylated peptide 131-151 and incubated with SYTOX Green and with or without 100 ng/mL PMA. Graphs show individual values and means of relative SYTOX fluorescence after 2 h incubation **(A)** or 2 and 4 h incubation **(B)**, respectively, normalized to starting values. One symbol represents one mouse. *n* = 3–8.

P140 has been previously shown to be active under conditions of increased autophagic flux (11, 15). Autophagy is activated in response to nutrient deprivation in almost all cell types. We therefore analyzed the effect of P140 in starved C3H mice. Again, NET formation was not significantly influenced upon injection of P140, ScP140, or non-phosphorylated peptide 131-51, as determined by plate reader-based fluorescence assay (Figure 4A) and morphology of immunofluorescence images of isolated mouse neutrophils (not shown).

So far, none of our data indicated that the ameliorative effect of P140/Lupuzor™ observed in patients with SLE and lupus mice could be related to the inhibition of NET formation. However, we had not yet looked at the influence of P140 under conditions of established lupus disease. We therefore analyzed NET formation in P140- and control peptide-treated MRL/lpr mice. Again no differences could be seen between MRL/lpr mice treated with P140, ScP140, or 131-151 peptides (Figure 4B).

**Figure 5:**
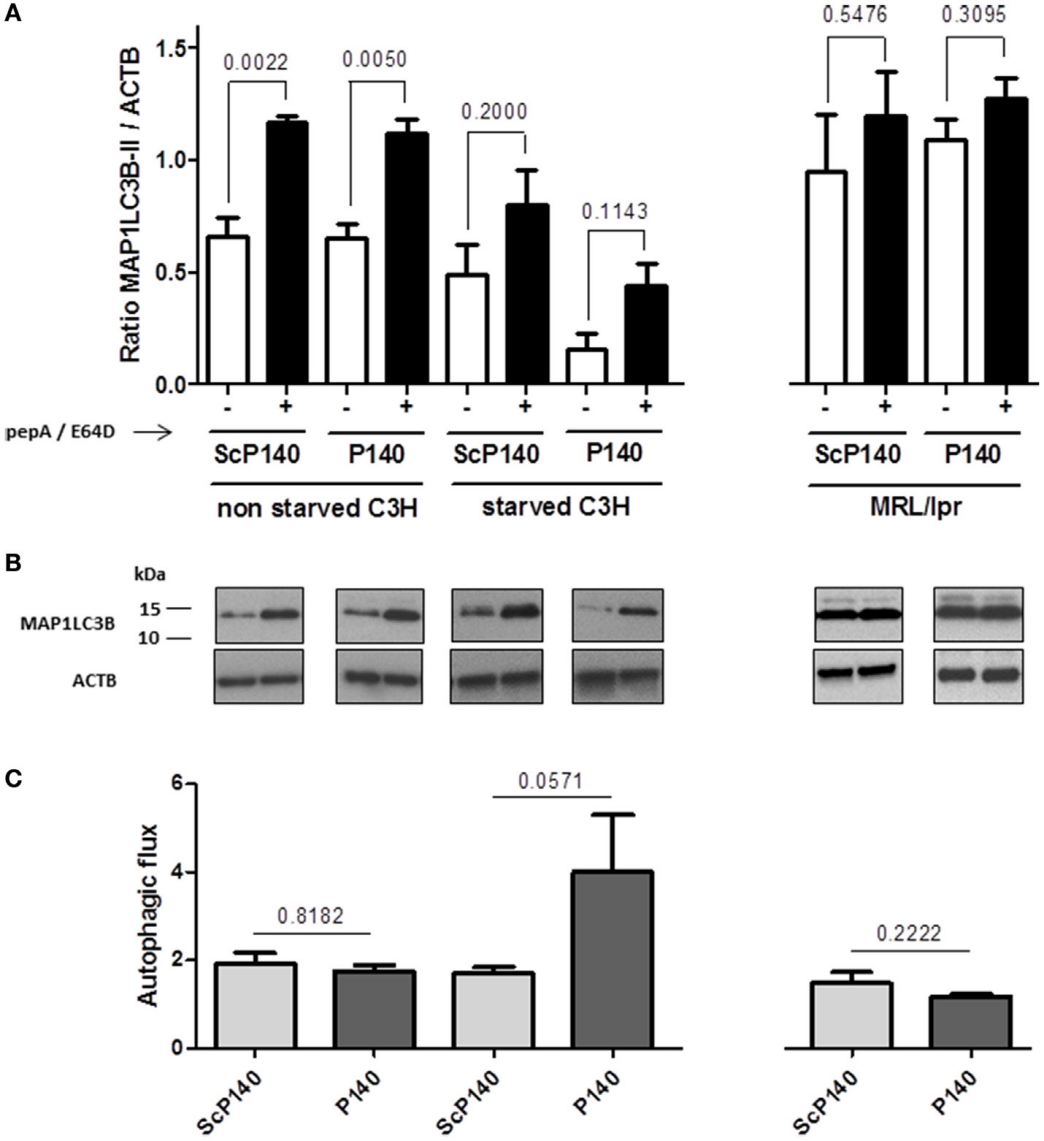
**Effect of P140 peptide on autophagic flux evaluated by measuring MAP1LC3B-II levels in total splenocytes from non-starved mice and starved C3H mice and MRL/lpr mice**. Cell lysates were resolved by SDS-PAGE, transferred onto polyvinylidene difluoride membranes before staining with anti-microtubule-associated-protein light chain 3b (MAP1LC3B) antibodies. When indicated, cells were treated (+) or not (−) during the last 4 h of the culture with 5 µg/mL pepstatin A and 5 µg/mL E64d to block lysosomal degradation. **(A)** MAP1LC3B-II levels were evaluated by densitometry and normalized to ACTB/β-actin. Histogram bars represent the means of individual experiments with SEM. *p < 0.05, using a Mann–Whitney *U* test to compare the data obtained in the presence or absence of protease inhibitors. **(B)** Representative immunoblot. ACTB was used as loading controls. **(C)** Autophagic flux as measured by comparing values of MAP1LC3B-II in the presence of lysosomal protease inhibitors divided by values of MAP1LC3B-II in the absence of lysosomal protease inhibitors. Histogram bars represent the means of individual experiments with SEM. *p < 0.05 using a Mann–Whitney test between the untreated and treated mice, in each condition. The groups were constituted of *n* = 6 for non-starved C3H mice, *n* = 4 for starved C3H mice, and *n* = 5 for MRL/lpr mice.

**Figure 6:**
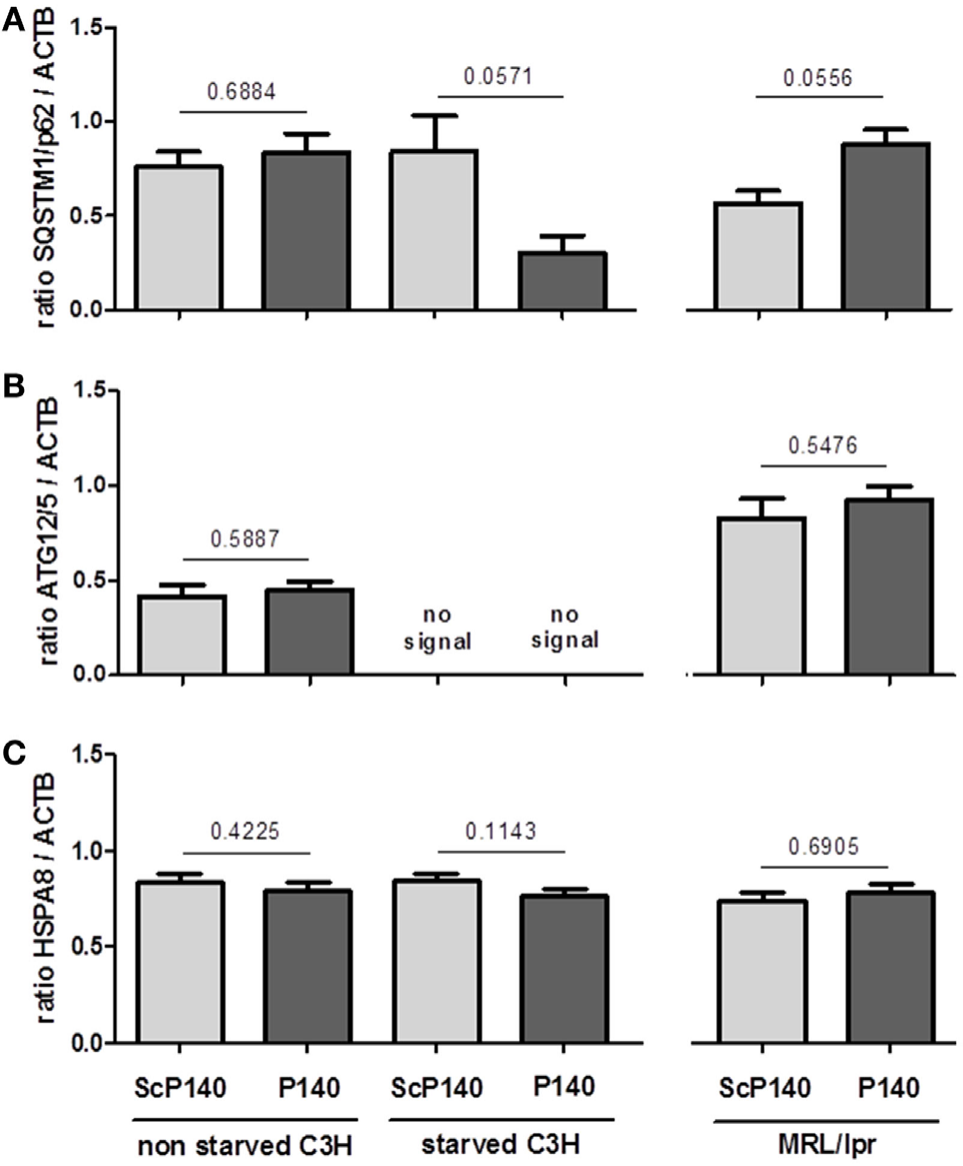
**Effect of P140 peptide on autophagy evaluated by measuring SQSTM1, ATG12/5, and HSPA8 levels in total splenocytes from non-starved mice, starved C3H mice, and MRL/lpr mice**. Three markers were followed, namely, **(A)** SQSTM1, **(B)** ATG12/5, and **(C)** HSPA8. The groups were constituted of *n* = 6 for non-starved C3H mice, *n* = 4 for starved C3H mice, and *n* = 5 for MRL/lpr mice. Histogram bars represent the means of individual experiments with SEM. *p < 0.05 using a Mann–Whitney test between the untreated and treated mice, in each condition.

The above data show no obvious evidence that the P140 peptide might directly influence neutrophils to undergo NET formation. We then investigated further the effect of P140 peptide on autophagy processes. The autophagic flux was measured by visualizing the expression of a well-established autophagy marker, namely, ATG8/MAP1LC3B, in calibrated conditions, in the presence or absence of lysosome proteases E64d and pepstatin A as described (25). We confirmed the existence of an active autophagic flux in total splenocytes of normal C3H mice, which however, was not altered by P140 peptide (Figures 5A–C, left). In spleen cells from starved C3H mice, however, no active flux was detectable and as expected, therefore, there was no significant effect of P140 peptide (Figures 5A–C). The same result as shown with cells from starved C3H mice was observed in MRL/lpr (activated) splenocytes (Figures 5A–C, right). For the same reasons, no statistically significant effect of P140 peptide was visualized when other markers of macroautophagy and CMA, such as ATG12/5 (that participates to the autophagosome formation), the autophagy substrate SQSTM1/p62, and the heat shock protein HSPA8, were followed in the whole splenocyte population (Figures 6A–C).

The results generated with normal C3H splenocytes, in which autophagic flux is active and showing no effect of P140 peptide, probably result from the fact that in normal (unstressed) cells, the peptide entry notably differs from the one it uses in activated/stressed cells and could not reach the cell compartment where it exerts its function (11).

**Figure 7:**
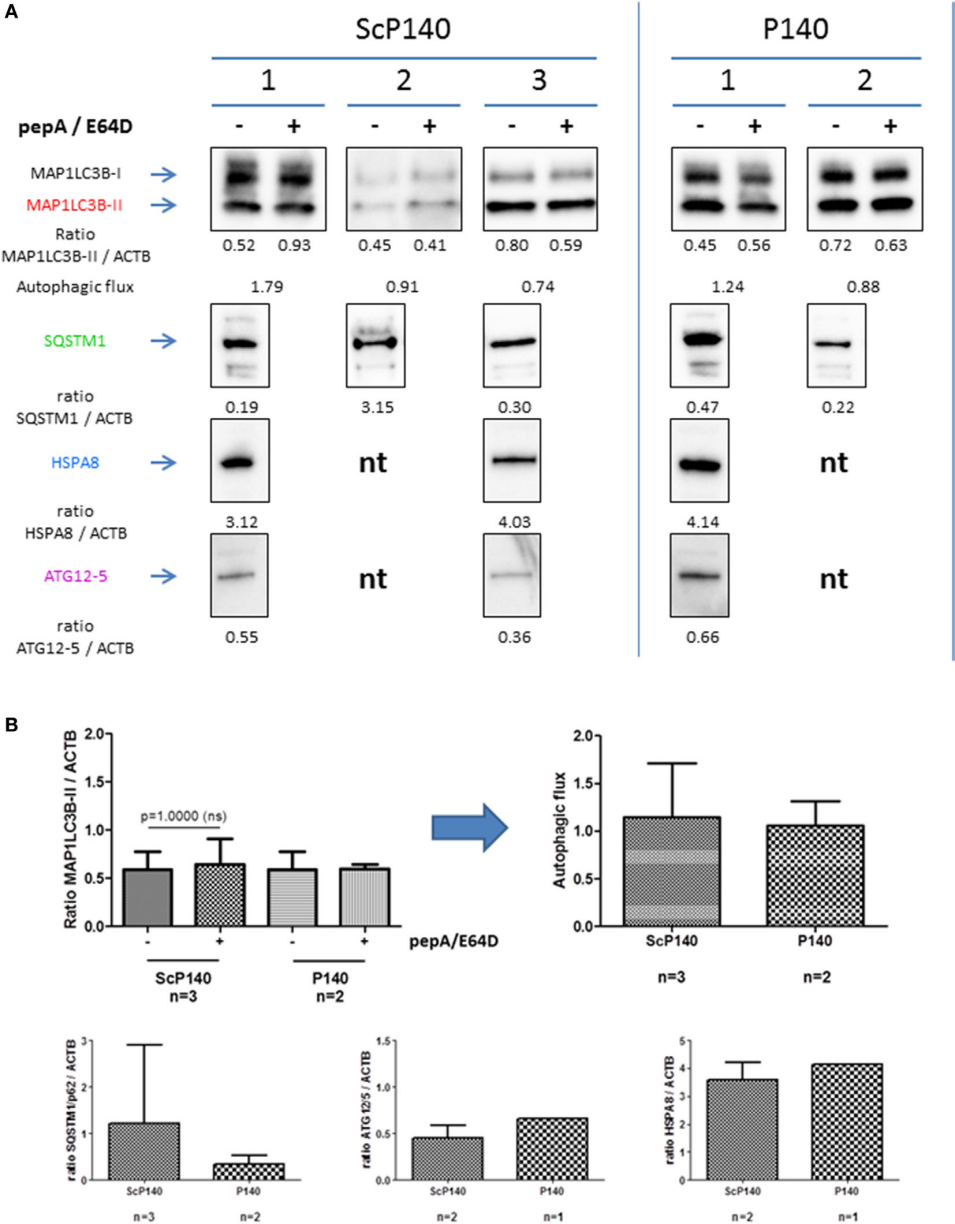
**Effect of P140 on autophagy in isolated splenic neutrophils**. Similar experiments were performed as in Figures 5 and 6, but with isolated splenic neutrophils instead of total splenoctes. **(A)** Immunoblots of microtubule-associated-protein light chain 3b (MAP1LC3B), SQSTM1, HSPA8, and ATG12/5 antibodies with three independent mice for control peptide scrambled P140 (ScP140) and two for P140 peptide. The expression levels were quantified by densitometry and normalized to ACTB/β-actin. For MAP1LC3B-II, autophagic flux was measured by dividing the values of MAP1LC3B-II with lysosomal protein inhibitor by those of MAP1LC3B-II without lysosomal protein inhibitor. **(B)** Histogram representation of immunoblots from Figure 7A. nt, not tested.

The absence of P140 effect in total splenocytes from 16-week-old diseased MRL/lpr mice contrasts with earlier results generated with B cells that were purified from the spleen of young (8-week-old) MRL/lpr mice (15). In B cells, P140 could effectively reduce the excessive autophagic flux. To investigate if P140 influences autophagy directly in neutrophils, we isolated splenic neutrophils from MRL/lpr mice treated with either 100 µg P140 or ScP140 (Figure 7). Interestingly, and in contrast to what was previously shown in lymphocytes (11, 15), autophagic flux was not activated in neutrophils from diseased MRL/lpr mice. Accordingly, P140 did not affect expression of autophagy markers as compared to ScP140.

## Discussion

Many aspects of neutrophil biology have been implicated in the pathogenesis of SLE (4). Especially the formation of NETs has been in the center of research during the last years (26–30). Typical during PMA-induced NETosis is the generation of many vesicles before plasma membrane rupture (16, 31). These vesicles have a double phospholipid bilayer and are believed to originate from the nuclear envelope, which disintegrates during NET formation. Finally, but still before plasma membrane permeabilization, nuclear chromatin decondenses and mixes with the contents of the granules; this is essential for formation of functional NETs. These arguments led us to examine if P140/Lupuzor™, a molecule that was shown to modulate autophagy in lymphocytes, could also alter NET formation or other aspects of neutrophil function, such as ROS production and the release of inflammatory mediators, which are important in both certain kinds of NET formation and the pathogenesis of lupus (31–33).

At this stage, however, our experimental data do not allow us to endorse this idea, at least in the experimental setting used in this study (namely, with diseased lupus mice). Treatment with P140 did not induce production by PMNs of ROS, cytokines, or chemokines and did not modulate NET formation in *in vitro and in vivo* assays. Consequently, a connection between CMA or macroautophagy and neutrophil functions cannot be drawn, in contrast to B lymphocytes, in which P140 modulates autophagy (11, 34). Several possible explanations can be put forward to explain this lack of effect.

First, and most importantly, we did not observe an increase of autophagic flux in neutrophils from diseased mice, which explains the lack of P140 activity in this cell type, because P140 is active under conditions of autophagic flux only. Second, no active flux in splenocytes of starved C3H mice could be visualized. It is widely accepted that autophagy is activated in response to nutrient deprivation in a variety of cell types. However, it has to be mentioned that when animals are starved, effects are more particularly observed in organs like the liver as compared to immune cells, and this feature could explain our results. Third, the *in vivo* administration protocol of P140 chosen in the present work could have influenced the results. An effect of P140 on autophagy markers in MRL/lpr splenocytes was visualized 5 days after a single injection of P140. This optimized protocol, however, could not be used with splenic neutrophils due to their short life expectancy and higher turn-over in the spleen. Since our experiments were focused on neutrophils, we decided to apply P140 a second time, 1 day before the isolation of neutrophils. It is possible that P140 administered under this alternative protocol we were obliged to apply, would have also no effect in lymphocytes.

Although P140 does not seem to directly influence NET formation, an indirect effect cannot be excluded. Patients with SLE show an increased rate of spontaneous NET formation and this phenotype is likely to be associated with the inflammatory status of the patients. Amelioration of the disease may in turn decrease the pre-activation of neutrophils resulting in less reactive neutrophils and reduced NET formation. To this regard, it is interesting to mention that compared to CBA/J control mice, HSPA8, a receptor of P140, is overexpressed at the cell surface of CD11b^+^ Gr-1^+^ granulocytes collected from the spleen of MRL/lpr mice (15). Upon P140 intravenous administration, granulocytes, as also monocytes and lymphocyte subsets that are over-represented in the peripheral blood of MRL/lpr mice, egress from the blood. Noticeably, contrary to other white blood cells subsets, which recolonize the blood in a few days, granulocytes remain at their basal level for at least 10 days before reappearing in the peripheral blood of P140-treated MRL/lpr mice (Schall et al., unpublished). These findings highlight the fact that P140 might be an attractive tool to target lupus neutrophils.

Since autophagy is often upregulated in response to cellular stress, further studies need to be conducted to analyze the influence of NET formation under such conditions.

## Ethics Statement

All experiments were approved by Regierung von Unterfranken, Germany, and Comité Régional d’Ethique en Matière d’Expérimentation Animale de Strasbourg. All subjects gave written informed consent.

## Author Contributions

CM planned and performed *in vitro* and *in vivo* experiments on NET formation, conducted data analysis, and drafted the manuscript. NS performed the experiments on autophagy and conducted data analysis. BG and FM performed experiments on autophagy. JH and DK performed experiments on NET formation. MH and SM planned the experiments, provided scientific input, and wrote the manuscript.

## Conflict of Interest Statement

The authors declare that the research was conducted in the absence of any commercial or financial relationships that could be construed as a potential conflict of interest.
